# Associations between neighbourhood characteristics, alcohol use patterns, and physical activity in midlife - the Northern Finland Birth Cohort 1966

**DOI:** 10.1007/s00127-026-03162-9

**Published:** 2026-07-17

**Authors:** Nina Rautio, Veera Säynäjäkangas, Tiina Lankila, Soile Puhakka, Anna-Maiju Leinonen, Jordina Bigas i Reverter, Jussi Seppälä, Raija Korpelainen, Markku Timonen, Jonna Levola, Jouko Miettunen

**Affiliations:** 1 Research Unit of Population Health, Faculty of Medicine, University of Oulu, P.O. Box 5000, FIN-90014 Oulu, Finland; 2 Medical Research Center Oulu, Oulu University Hospital and University of Oulu, Oulu, Finland; 3 Department of Psychiatry, Oulu University Hospital, Oulu, Finland; 4 Geography Research Unit, University of Oulu, Oulu, Finland; 5 Department of Sports and Exercise Medicine, Oulu Deaconess Institute Foundation sr, Oulu, Finland; 6 Research Unit of Health Sciences and Technology, University of Oulu, Oulu, Finland; 7 Social Insurance Institution of Finland, Kuopio, Finland; 8 Department of Psychiatry, University of Helsinki and Helsinki University Hospital, Helsinki, Finland

**Keywords:** Alcohol use, Urban, Rural, Neighbourhood, Physical activity

## Abstract

**Purpose:**

Despite extensive research, links between individuals’ environments and alcohol use remain inconsistent. This has renewed interest in neighbourhood determinants of alcohol use. We examined whether neighbourhood characteristics are related to alcohol use in urban and rural areas, and whether physical activity modifies associations.

**Methods:**

Cross-sectional study utilised 46-year follow-up data from the population-based Northern Finland Birth Cohort 1966 (*n* = 6,655). Alcohol use was classified into abstinence, moderate (> 0 ≤ 30/20 g/day men/women), and heavy use (> 30/20 g/day). Neighbourhood characteristics were derived from national geographic data sources using residential coordinates. Physical activity was assessed through self-reports and accelerometry. Multinomial logistic regression analyses were conducted to examine relationships between neighbourhood characteristics and alcohol use in urban and rural areas, adjusting for socio-demographic factors and physical activity.

**Results:**

Heavy use was common in urban areas, while abstinence was more common in rural areas. In urban areas, greater proportions of rented housing, more bus stops and grocery stores, and longer pedestrian/cycle paths were associated with heavy alcohol use. Greener environments were associated with lower levels of heavy use. In rural areas, a higher proportion of rented housing was associated with higher likelihood of abstinence. In both areas, living in more advantaged neighbourhoods was associated with lower likelihood of abstinence. Physical activity generally did not modify associations between neighbourhood characteristics and alcohol use.

**Conclusion:**

Neighbourhood characteristics are associated with alcohol use among middle-aged individuals, showing different patterns in urban and rural areas. These findings highlight the importance of considering neighbourhood characteristics in public health strategies for reducing alcohol use.

## Introduction

Alcohol use accounts for a significant portion of social harms, overall disease burden, and mortality globally [1]. Despite these concerns, more than 75% of the population in high-income European regions used alcohol in 2019 [1]. In 2023, 48% of Finnish men and 22% of women who consumed alcohol did so weekly [2]. Even though alcohol use has declined in recent years in Finland and in Europe [1, 2], understanding the determinants of use is essential to develop effective public health strategies.

Alcohol use varies geographically both across and within countries, being influenced by a multifaceted mix of regional demographic, social, cultural, and economic factors, including prevailing local attitudes towards drinking [3, 4]. In addition, neighbourhood characteristics may shape alcohol use patterns. These patterns vary with population density, reflecting urban and rural use [5]. In one scoping review, nearly 60% of the included studies found that heavy or high-risk alcohol use was more common in rural than in urban communities, almost 18% reported it to be more likely in urban communities, and about 14% found no difference [3]. In men, weekly drinking is reported to be more common in urban areas, while the proportion of daily and problem drinking (e.g., drinking in the morning and being unable to work) among those who drink weekly is higher in rural areas [6].

Differences in alcohol use between rural and urban areas may be influenced by the availability of alcohol. Studies have shown that higher alcohol outlet density is associated with higher alcohol consumption, while greater distance from the nearest alcohol outlet is associated with lower use [7–10]. The density of alcohol retail outlets is closely tied to the broader built environment. The built environment refers to a neighbourhood’s physical characteristics, such as housing types, roads, footpaths, transportation networks, shops, parks, and public spaces [11]. People living in poorer neighbourhoods (i.e., with a higher percentage of buildings in dilapidated condition) are up to 1.5 times more likely to report heavy drinking than those in better-built areas [12]. The proportion of single detached houses and townhouses is also positively associated with alcohol expenditure [10]. Alcohol use is influenced not only by characteristics of the built environment but also by proximity to green spaces. Among adolescents and young adults, greater residential greenness is associated with lower odds of frequent binge drinking, i.e., consuming a large amount of alcohol in a short period of time [13].

The built and natural environments also interact with the social environment to shape patterns of alcohol use and reflect area-based socioeconomic disparities. A systematic review of area-level disadvantage found mixed evidence on alcohol-related outcomes: of 89 effect estimates, 18% supported the disadvantage hypothesis, almost 14% indicated higher alcohol use in affluent neighbourhoods, and 68% showed no association [14].

Recent research has linked physical activity to behavioural factors including alcohol use and environmental determinants such as infrastructure and accessibility. A systematic review reported a positive association between physical activity and alcohol use in early adulthood [15, 16]. On the other hand, healthy behaviours tend to cluster somewhat [17]. Furthermore, another systematic review found that higher levels of physical activity were mainly associated with the development of new infrastructure for walking, cycling, and public transit, enhancements to parks and playgrounds, and objectively assessed accessibility [18]. Examining neighbourhood characteristics, physical activity, and alcohol use is vital from a public health perspective.

This population-based study investigates associations between the built and natural characteristics of residential environments, area-level socioeconomic disparities, and alcohol use patterns in midlife in urban and rural areas, and analyses whether such associations are modified by physical activity.

## Methods

### Study population

**Fig. 1:**
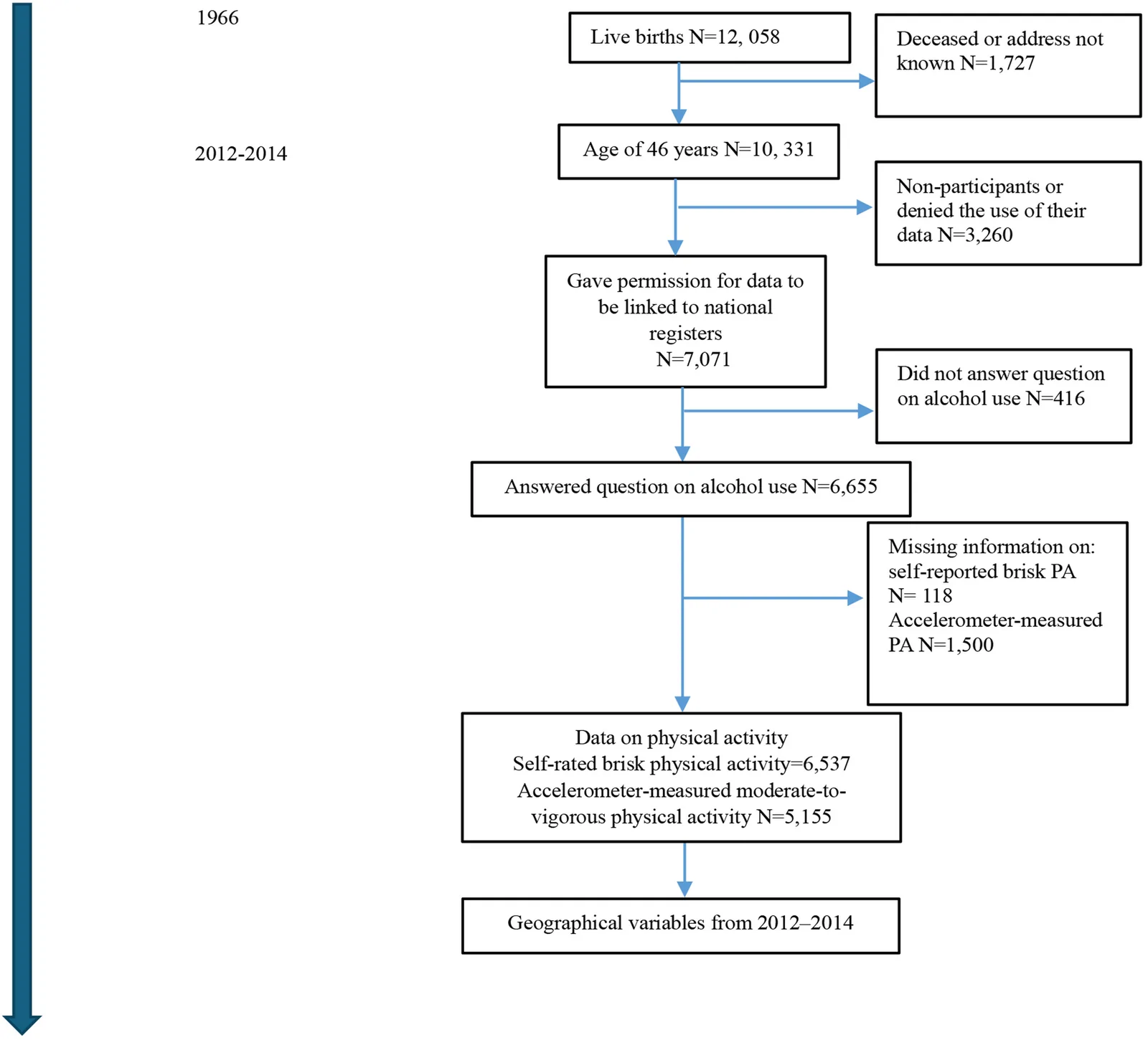
Flowchart of study population

We utilised data from the Northern Finland Birth Cohort 1966 (NFBC1966) study, which followed participants longitudinally using a population-based design [19]. In 1966, 12,058 liveborn children from the regions of Oulu and Lapland (covering 96.3% of all births) were enrolled in the NFBC1966 study (Fig. 1).

This cross-sectional study utilised data from the most recent collection, obtained via online or paper questionnaires and clinical assessments conducted during the 46-year follow-up period from 2012 to 2014. Details regarding the follow-up process, including attrition and missing data, have been previously reported [20]. The study was approved by the Ethical Committee of the Northern Ostrobothnia Hospital District 94/2011 (12.12.2011), and written informed consent was obtained from all participants.

Out of 10,331 eligible participants, 7,071 (68.4%) consented to data usage and linkage with national registers, while 6,655 (64.4%) provided information on alcohol use. All those with information on alcohol use were included in this study (Fig. 1). Self-rated brisk physical activity and accelerometer-measured physical activity data were available for 6,537 and 5,155 individuals, respectively.

### Alcohol use

Data on alcohol use were collected through a questionnaire, which included items on both frequency and quantity [21]. Frequency was assessed using a 10-point scale ranging from 1 (never) to 10 (daily). Typical quantities consumed were assessed separately for beer, wine, and spirits. For beer, participants reported the number of 0.33-litre bottles typically consumed, using a 9-point scale from 1 (none) to 9 (15 bottles or more). Wine consumption was recorded in terms of glasses (16 cl) or 0.75-litre bottles, and spirits were measured in 4 cl shots or 0.5-litre bottles. Similar 9-point scales were used to assess quantities for both wine and spirits. For calculation purposes, we considered one bottle of beer and one shot of spirits as one standard alcohol unit (12 g of ethanol), and wine as having an alcohol content of 13% with one glass of wine containing 16.4 g of ethanol. Based on responses, daily ethanol use was estimated by multiplying the reported frequency of use by the typical quantity used. Participants were then classified based on their alcohol use [22]. Heavy alcohol use was defined as consuming more than 30 g/day for men and more than 20 g/day for women. Abstainers reported no consumption, and the rest were classified as moderate users [22], who served as the reference category in statistical analyses.

### Urban and rural areas

Urban and rural areas were classified using a geospatial method based on nationwide 250 × 250 m statistical grid data from the year 2010, available from the Finnish Environment Institute [23]. Finland’s areas were classified into seven different categories: (1) inner urban area, (2) outer urban area, (3) peri-urban area, (4) local centres in rural areas, (5) rural areas close to urban areas, (6) rural heartland areas, and (7) sparsely populated rural areas [23, 24]. For this study Categories 1–3 were combined as urban areas and Categories 4–7 as rural areas. Participants were assigned to urban/rural areas based on residential coordinates.

### Neighbourhood characteristics

Neighbourhood characteristics were derived using ArcMap ArcGIS Pro 2.1 [25] based on participants’ residential coordinates. A circular buffer with a 1 km radius was applied around each residence to represent the surrounding neighbourhood. The distance between each participant’s home and certain destinations was also determined. Proportions of working‑aged people, rented housing, and number of grocery stores and restaurants, as well as characteristics included in the neighbourhood advantage score, were derived from the Finnish Community Structure Follow‑up System (FCSFS) and based on tabular spatial datasets constructed from 250 m × 250 m grid cells [26] whose midpoint was within the circular buffer. The distances from a participant’s home to the nearest local service centre and grocery store by road were calculated using the midpoints of the closest 1 km statistical grid or the midpoint of the nearest 250 m x 250 m grid cell and data from the Finnish National Road and Street Database [27]. Data on bus stops and cycle/pedestrian paths were obtained from the Finnish National Road and Street Database [27], with bus stops counted and path lengths summed within a 1 km buffer. The number of sports facilities was obtained from Lipas, the national database of sports facilities [28], and calculated within a 1 km radius. The straight-line distance in kilometres to the nearest park or forest was calculated using the CORINE Land Cover vector dataset (minimum mapping unit: 25 hectares) from the Finnish Environment Institute [23]. Residential greenness was assessed using the Normalised Difference Vegetation Index (NDVI) [29], calculated within a 1 km radius. Neighbourhood data from 2012 to 2014 were linked to participants’ responses on alcohol use, matched by the date of response. Environmental variables were available from the year of response, except data on bus stops and cycle/pedestrian paths and sports facilities which were from 2014, NDVI data which were from 2013 to 2015, and parks and forest data which were from 2012. Residence start and end dates were obtained from the Digital and Population Data Services Agency [30]. If the exact start date was missing but information on the month and year was available, the last day of the previous month was used as the residence start date.

*Proportion of working-aged (18–64) people* was based on the age distribution of the population [26], and the proportion of working-aged individuals was calculated from the total population. *Proportion of rented housing* [26] was calculated as the proportion of all housing, primarily for permanently occupied dwellings (indicated by ownership or other arrangements such as right-of-occupancy dwelling, life annuity housing, kinship-based occupancy, or unknown tenure status). *Distance to the nearest local service centre* [27] was calculated as the distance by road from the participant’s residence to the closest 1 km statistical grid cell with a shop and a restaurant. *Distance to the nearest grocery store* [27] was calculated as the distance by road between the participant’s residence and the midpoint of the nearest statistical 250 m × 250 m grid cell containing a grocery store. *Number of grocery stores and restaurants* [26] was used to determine the availability of local services. Grocery stores were counted as retail food outlets, and restaurants were defined as restaurant buildings, including canteens. For *bus stops and cycle/pedestrian paths* [27], the number of bus stops was determined within a 1 km radius, while the total length (in kilometres) of cycle and pedestrian paths was calculated within the same buffer zone. *Number of sports facilities* [28] did not include maintenance buildings. *Parks and forests* [23] were quantified by measuring the straight-line distance (in kilometres) from the participant’s residence to the nearest park or forest. Classification codes for green urban areas (1.4.1 green urban areas) and forests (3.1.1 broad-leaved forest; 3.1.2 coniferous forest; 3.1.3 mixed forest) were included from CORINE. The level of *residential greenness* within a neighbourhood was assessed using the Normalised Difference Vegetation Index (NDVI) [29]. NDVI was calculated within a 1 km buffer surrounding each residence and the mean value of this buffer was used to represent residential greenness. NDVI values were derived from information on reflected near-infrared radiation (NIR) and reflected visible light (VIS) using the formula (NIR − VIS) / (NIR + VIS) and reported within a range of − 1.0 to + 1.0, with higher values indicating more abundant and denser vegetation [31]. Negative NDVI values were set to zero. The index was calculated using Landsat 8 (L8) satellite imagery (resolution 30 × 30 m) provided by the United States Geological Survey (USGS) [32]. Images with less than 10% cloud cover were selected, and the months of June to July (2013–2015) were used in the calculation. *Neighbourhood advantage score* [26] considered employment rate, proportion of the workforce with higher education, and median household income. Due to the data structure, unemployment rates could not be determined. The employment rate was calculated by dividing the number of people in the workforce by the area’s population aged 18 to 74, and the proportion of the workforce with higher education was calculated by dividing the proportion of the workforce with higher education by the total workforce. A z-score (mean = 0, SD = 1) was then calculated for each of the three variables by subtracting the mean of each variable from the raw score and dividing the result by the variable’s standard deviation. Finally, the neighbourhood advantage score was calculated as the average of all three z-scores.

### Self-reported and accelerometer-measured physical activity

Self-reported physical activity was assessed through a question on frequency (ranging from once a month or less often (0) to daily (7)) and duration (from 0 (0) to over 1.5 h (90 min) per day) of brisk physical activity during leisure time [33]. Brisk physical activity was defined as an activity that caused at least some sweating and breathlessness. The weekly amount of brisk physical activity in minutes was calculated as frequency x duration and divided by seven to determine average daily physical activity (min/day).

In clinical examinations, participants were required to wear a wrist-worn waterproof Polar Active monitor (Polar Electro Oy, Kempele, Finland). They were instructed to wear the monitor on their non-dominant hand for at least two weeks (including during sleep) and maintain their normal daily routines. They did not receive any feedback from the accelerometer during data collection. Participants with at least four valid days (wear time of at least 600 min/day during waking hours) were included in the analyses [34, 35]. The Polar Active monitor calculated estimated metabolic equivalent of task (MET) values every 30 s based on daily activity levels [36]. The data were classified into five levels according to the cut-offs of the device manufacturer: sedentary behaviour (< 2 METs), light (2-3.5 METs), moderate (3.5-5 METs), vigorous (5–8 METs), and very vigorous (≥ 8 METs) activity. Daily average durations (min/day) of moderate, vigorous, and very vigorous [35] activity were calculated for each participant and were combined into minutes per day of moderate-to-vigorous physical activity. Polar Active has been shown to correlate with the double-labelled water technique when measuring energy expenditure during exercise in free living conditions [37].

### Covariates

Sex, marital status, and educational attainment were considered as covariates based on earlier research [38, 39] and preliminary analyses. Marital status was classified into two groups: married/cohabiting/registered partnership and single/divorced/widowed. Educational level was evaluated using two questionnaire items covering both general and vocational education and classified into three levels: basic (≤ 9 years of schooling with no vocational training or only short courses), secondary (completion of vocational school, general upper secondary school, and/or matriculation examination), and tertiary (polytechnic or university degree).

### Statistical analyses

Descriptive analyses were conducted to compare background and neighbourhood characteristics between urban and rural areas. Due to the collinearity of the neighbourhood characteristics, each characteristic was included in a separate multinomial logistic regression model, and all analyses were stratified by urban and rural areas using the three categories of alcohol use (moderate (reference), abstainer, and heavy use) as the outcome (Model 1). Model 2 was adjusted for sex, marital status, and educational attainment. Model 3 was adjusted for self-rated brisk physical activity, sex, marital status, and educational attainment, and Model 4 additionally incorporated accelerometer-based moderate-to-vigorous physical activity along with sociodemographic factors.

## Results

The characteristics of the study population and neighbourhoods in urban and rural areas are presented in Table 1. Most participants were women (54.3%), married or in a de facto relationship (77.5%), and had secondary education (69.2%). Heavy alcohol use was more common among urban than rural residents, whereas rural residents were more likely to abstain from alcohol use. Consumption of beer and wine was higher in urban areas than in rural areas, whereas consumption of spirits showed the opposite pattern (Table 1). Mean self-rated brisk physical activity was 15.7 (SD 17.1) and accelerometer-measured moderate-to-vigorous physical activity 68.8 (SD 35.0) minutes/day. There were differences in most neighbourhood characteristics between urban and rural areas (Table 1).

**Table 1:** Characteristics of the study population and neighbourhood in urban and rural areas (*n* = 6,655)

Characteristics	Total (*n* = 6655)	Urban (*n* = 4255)	Rural (*n* = 2400)
**Sex**			
Male	3042 (45.7)	1885 (44.3)	1157 (48.2)
Female	3613 (54.3)	2370 (55.7)	1243 (51.8)
**Marital status**			
Married/de facto relationship	5140 (77.5)	3200 (75.4)	1940 (81.3)
Single/divorced/widowed	1493 (22.5)	1046 (24.6)	447 (18.7)
**Education**			
Primary	278 (4.2)	128 (3.0)	150 (6.3)
Secondary	4597 (69.2)	2746 (64.6)	1851 (77.4)
Tertiary	1770 (26.6)	1378 (32.4)	392 (16.3)
**Alcohol use**			
Abstainer	648 (9.7)	332 (7.8)	316 (13.2)
Moderate	5253 (79.0)	3414 (80.2)	1839 (76.6)
Heavy	754 (11.3)	509 (12.0)	245 (10.2)
**Alcohol use by beverage type**	Mean (SD)	Mean (SD)	Mean (SD)
Beer (g/day)	7.62 (15.72)	8.07 (16.23)	6.84 (14.75)
Wine (g/day)	1.85 (4.42)	2.23 (4.96)	1.17 (3.14)
Spirits (g/day)	2.03 (8.55)	1.86 (8.24)	2.33 (9.08)
**Physical activity**			
Self-rated brisk (min/day)	15.74 (17.13)	16.64 (17.37)	14.12 (16.57)
Accelerometer measured moderate- to-vigorous (min/day)	68.81 (34.95)	68.02 (31.88)	70.27 (39.98)
**Neighbourhood**			
Proportion of working-aged people	0.61 (0.07)	0.63 (0.05)	0.57 (0.09)
Proportion of rented housing	0.31 (0.16)	0.30 (0.16)	0.33 (0.17)
Distance to closest local service centre (km)	5.50 (7.85)	2.86 (2.35)	10.18 (11.26)
Distance to closest grocery store (km)	3.04 (5.37)	1.25 (1.36)	6.21 (7.81)
Number of grocery stores	2.04 (2.79)	2.37 (3.07)	1.12 (1.47)
Number of restaurants	0.70 (1.49)	0.86 (1.71)	0.42 (0.89)
Number of bus stops	4.21 (7.99)	6.20 (9.34)	0.69 (1.74)
Pedestrian/cycle paths (km)	11.18 (10.25)	15.58 (9.90)	3.38 (4.69)
Number of sports facilities	14.53 (16.68)	18.58 (17.95)	7.37 (10.96)
Distance to closest park (km)	19.80 (28.0)	6.49 (13.75)	43.39 (31.08)
Distance to closest forest (km)	0.47 (0.57)	0.58 (0.63)	0.26 (0.37)
Residential greenness	0.42 (0.15)	0.39 (0.15)	0.48 (0.13)
Neighbourhood advantage score	0.16 (0.68)	0.34 (0.70)	-1.16 (0.80)

### Associations between urban neighbourhood characteristics and alcohol use patterns

After adjusting for sex, marital status, and educational attainment, a higher proportion of working-age people (OR = 0.02, 95% CI 0.00, 0.14) was associated with a lower likelihood of abstinence, though the estimate was close to the null. Living in a more advantaged neighbourhood (OR = 0.74, 95% CI 0.62, 0.87) was also associated with a lower likelihood of abstinence. In contrast, longer distance to a grocery store (OR = 1.11, 95% CI 1.04, 1.20) was associated with a higher likelihood of abstinence (Table 2).

A higher proportion of working-age people in the neighbourhood (OR = 13.91, 95% CI 2.30, 84.26) was associated with a greater likelihood of heavy alcohol use after adjustments; however, the confidence intervals were wide. A similar association was observed for higher proportion of rented housing in the neighbourhood (OR = 2.27, 95% CI 1.21, 4.27). Greater distance to a grocery store was associated with lower odds of heavy alcohol use (OR = 0.90, 95% CI 0.83, 0.98), while a higher density of grocery stores was associated with a higher risk of heavy alcohol use (OR = 1.05, 95% CI 1.02,1.08). Higher number of bus stops (OR = 1.01, 95% 1.00, 1.02) and greater total length of pedestrian/cycle paths (OR = 1.02, 95% CI 1.01, 1.03) were associated with a higher risk of heavy alcohol use, but the associations were very small in magnitude.

Greater distance to the nearest forest was associated with higher odds of heavy alcohol use (OR = 1.19, 95% CI 1.03, 1.38), whereas a higher level of residential greenness was associated with lower risk of heavy use (OR = 0.41, 95% CI 0.22, 0.77). In addition, living in a more advantaged neighbourhood was associated with a higher likelihood of heavy use (OR 1.15, 95% CI 1.00, 1.32). The results remained the same after separate adjustments for self-rated brisk physical activity and accelerometer-measured moderate-to-vigorous physical activity.

### Association between rural neighbourhood characteristics and alcohol use patterns

In rural areas, a higher proportion of working-age people in the neighbourhood was associated with a lower likelihood of abstinence after adjusting for sex, marital status, and educational attainment (OR = 0.03, 95% CI 0.01, 0.13), though the effect was weak (Table 3). Conversely, a higher proportion of rented housing in a neighbourhood was related to a higher likelihood of abstinence (OR = 4.17, 95% CI 1.54, 11.32). Consistent with findings in urban areas, living in a more advantaged neighbourhood was associated with a lower likelihood of abstinence. Results were robust after adjusting for self-rated brisk physical activity. However, after adjustment for accelerometer-measured moderate-to-vigorous physical activity, the association between the proportion of rented housing and abstinence attenuated to non-significant (OR = 2.33, 95% CI 0.75, 7.27) (Table 3).

**Table 2:** Associations between neighbourhood characteristics and alcohol use patterns (with moderate use as the reference) in middle-aged participants living in urban areas

Characteristics	Abstinence				Heavy use			
	Model 1^a^	Model 2^b^	Model 3^c^	Model 4^d^	Model 1^a^	Model 2^b^	Model 3^c^	Model 4^d^
Proportion of working-aged people	0.02 (0.00, 0.15)	0.02(0.00, 0.14)	0.02 (0.00, 0.18)	0.02 (0.00,0.24)	8.42 (1.52, 46.81)	13.91(2.30, 84.26)	15.51(2.51,95.68)	30.00 (3.68, 243.98)
Proportion of rented housing	0.85 (0.40, 1.78)	0.73 (0.34, 1.59)	0.79 (0.36, 1.74)	0.53 (0.22, 1.30)	2.09 (1.15, 3.80)	2.27 (1.21, 4.27)	2.26(1.19, 4.30)	2.27 (1.08, 4.78)
Distance to closest local service centre (km)	1.03 (0.98, 1.07)	1.03 (0.98, 1.08)	1.03 (0.98, 1.08)	1.03 (0.98, 1.09)	0.98 (0.94, 1.02)	0.98 (0.94, 1.02)	0.98 (0.94, 1.02)	0.97 (0.92, 1.02)
Distance to closest grocery store (km)	1.10 (1.03, 1.18)	1.11 (1.04, 1.20)	1.11 (1.04, 1.20)	1.12 (1.04, 1.21)	0.91 (0.84, 0.99)	0.90 (0.83, 0.98)	0.90 (0.83,0.98)	0.89 (0.80, 0.98)
Number of grocery stores	0.99 (0.95,1.03)	0.99 (0.95, 1.03)	1.00 (0.95, 1.04)	0.99 (0.94, 1.04)	1.04 (1.01, 1.07)	1.05 (1.02, 1.08)	1.04 (1.02,1.07)	1.06 (1.02, 1.09)
Number of restaurants	0.98 (0.92, 1.05)	0.98 (0.91, 1.06)	0.98 (0.92, 1.06)	0.97 (0.89, 1.06)	1.04 (0.99, 1.09)	1.04 (0.99, 1.10)	1.04 (0.99, 1.10)	1.04 (0.98, 1.10)
Number of bus stops	0.99 (0.97, 1.00)	0.99 (0.97, 1.00)	0.99 (0.97, 1.00)	0.98 (0.96, 1.00)	1.01 (0.99, 1.02)	1.01 (1.00, 1.02)	1.01 (1.00,1.02)	1.01 (1.00, 1.02)
Pedestrian/cycle paths (km)	0.99 (0.98, 1.01)	0.93 (0.98, 1.01)	0.99 (0.98, 1.01)	0.99 (0.98, 1.01)	1.02 (1.01. 1.024)	1.02 (1.01, 1.03)	1.02 (1.01,1.03)	1.02 (1.01, 1.03)
Number of sports facilities	1.00 (0.99, 1.00)	1.00 (0.99, 1.00)	1.00 (0.99, 1.00)	0.99 (0.99, 1.00)	1.01(1.00,1.01)	1.01 (1.01, 1.02)	1.01 (1.01, 1.02)	1.01 (1.00, 1.02)
Distance to closest park (km)	1.00 (1.00, 1.01)	1.00 (1.00, 1.01)	1.00 (0.99, 1.01)	1.00 (0.99, 1.01)	1.00 (0.99, 1.00)	1.00 (0.99, 1.00)	1.00 (0.99, 1.00)	1.00 (0.99, 1.01)
Distance to closest forest (km)	1.00 (0.84, 1.20)	1.01 (0.84, 1.21)	1.03 (0.85, 1.23)	0.96 (0.77, 1.20)	1.17 (1.02. 1.33)	1.19 (1.03, 1.38)	1.20 (1.04, 1.39)	1.19 (1.00, 1.40)
Residential greenness	1.53 (0.71, 3.31)	1.39 (0.63, 3.08)	1.38 (0.61, 3.11)	1.46 (0.58, 3.69)	0.50 (0.27, 0.91)	0.41 (0.22, 0.77)	0.37 (0.20, 0.69)	0.41 (0.20, 0.86)
Neighbourhood advantage score	0.72 (0.61, 0.84)	0.74 (0.62, 0.87)	0.73 (0.62, 0.86)	0.73 (0.61, 0.88)	1.09 (0.95,1.24)	1.15 (1.00, 1.32)	1.16 (1.01, 1.34)	1.23 (1.04, 1.45)

^a^ Model 1: crude (*N* = 4,086 − 4,255); ^b^ Model 2: adjusted for sex, marital status, and educational attainment (*N* = 4,076 − 4,262); ^c^ Model 3: adjusted for sex, marital status, educational attainment, and self-reported brisk physical activity (*N* = 4,009 − 4,173); ^d^ Model 4: adjusted for sex, marital status, educational attainment, and accelerometer-measured moderate-to-vigorous physical activity (*N* = 3,203-3,344)

**Table 3:** Associations between neighbourhood characteristics and alcohol use patterns (with moderate use as the reference) in middle-aged participants living in rural areas

Characteristics	Abstinence				Heavy use			
	Model 1^a^	Model 2^b^	Model 3^c^	Model 4^d^	Model 1^a^	Model 2^b^	Model 3^c^	Model 4^d^
Proportion of working-age people	0.03 (0.01, 0.11)	0.03 (0.01, 0.13)	0.03 (0.01, 0.13)	0.03 (0.01, 0.13)	3.25(0.82,12.88)	4.19 (1.01,17.36)	4.19 (0.97, 18.04)	2.60 (1.79, 3.72)
Proportion of rented housing	4.34(1.63,11.53)	4.17 (1.54, 11.32)	3.89 (1.42, 10.68)	2.33 (0.75, 7.27)	1.75 (0.59, 5.21)	1.47 (0.49, 4.44)	1.43 (0.46, 4.49)	0.85 (0.22, 3.35)
Distance to closest local service centre (km)	1.00 (0.99, 1.01)	1.00 (0.99, 1.01)	1.00 (0.99, 1.01)	1.01 (0.99, 1.02)	1.00 (0.99, 1.01)	1.00 (0.99, 1.01)	1.00 (0.99, 1.01)	0.99 (0.98, 1.01)
Distance to closest grocery store (km)	1.00 (0.99, 1.02)	1.00 (0.99, 1.0)	1.00 (0.99, 1.02)	1.01 (1.00, 1.03)	0.99 (0.98, 1.02)	1.00 (0.98, 1.01)	1.00 (0.98, 1.01)	0.99 (0.97, 1.02)
Number of grocery stores	0.99 (0.90, 1.11)	0.98 (0.89, 1.10)	1.00 (0.89, 1.11)	0.97 (0.86, 1.10)	1.04 (0.93, 1.16)	1.04 (0.93, 1.16)	1.04 (0.93, 1.17)	1.03 (0.90, 1.18)
Number of restaurants	0.99 (0.87, 1.13)	0.98 (0.86, 1.13)	0.99 (0.87, 1.14)	0.95 (0.81, 1.12)	0.98 (0.84, 1.14)	1.00 (0.85, 1.17)	1.00 (0.85, 1.18)	1.02 (0.85, 1.23)
Number of bus stops	0.99 (0.91, 1.07)	0.98 (0.91, 1.06)	0.99 (0.91, 1.06)	0.97 (0.89, 1.07)	1.04 (0.98, 1.10)	1.04 (0.97, 1.10)	1.04 (0.98, 1.11)	1.03 (0.95, 1.11)
Pedestrian/cyclist paths (km)	0.98 (0.95, 1.01)	0.98 (0.95, 1.00)	0.98 (0.95, 1.01)	0.98 (0.95, 1.01)	0.99 (0.96, 1.02)	0.91 (0.96, 1.02)	1.00 (0.97, 1.03)	0.99 (0.95, 1.02)
Number of sports facilities	0.99 (0.99, 1.01)	0.99 (0.98, 1.01)	0.99 (0.98, 1.01)	1.00 (0.98, 1.01)	0.99 (0.97, 1.00)	0.99 (0.97, 1.00)	0.99 (0.98, 1.00)	0.99 (0.97, 1.00)
Distance to closest park (km)	1.00 (0.99, 1.01)	1.00 (0.99, 1.01)	1.00 (1.00, 1.01)	1.00 (1.00, 1.01)	1.00 (0.99,1.00)	1.00 (0.99, 1.00)	1.00 (0.99, 1.00)	0.99 (0.99, 1.00)
Distance to closest forest (km)	1.01 (0.73, 1.39)	0.99 (0.72, 1.38)	1.04 (0.75, 1.44)	0.95 (0.65, 1.40)	0.76 (0.51, 1.14)	0.78 (0.52, 1.17)	0.82 (0.54, 1.23)	0.92 (0.57, 1.46)
Residential greenness	1.08 (0.43,2.72)	1.30 (0.50, 3.36)	1.17 (0.45, 3.04)	2.18 (0.70, 6.76)	2.22 (0.76, 6.47)	1.89 (0.64, 5.64)	1.77 (0.58, 5.36)	1.34 (0.36, 5.07)
Neighbourhood advantage score	0.81 (0.70, 0.95)	0.80 (0.69, 0.94)	0.82 (0.70, 0.96)	0.80 (0.67, 0.95)	0.86 (0.73, 1.01)	0.86 (0.72, 1.02)	0.87 (0.73, 1.04)	0.82 (0.66, 1.02)

^a^ Model 1: Crude (*N* = 1,321-2,400); ^b^ Model 2: adjusted for sex, marital status, and educational attainment (*N* = 1,314-2,383); ^c^ Model 3: adjusted for sex, marital status, educational attainment, and self-reported brisk physical activity (*N* = 1,284-2,338); ^d^ Model 4: adjusted for sex, marital status, educational attainment, and accelerometer-measured moderate-to-vigorous physical activity (*N* = 976-1,792); ^e^ Values are odds ratios and 95% confidence intervals

## Discussion

### Principal findings

This population-based study found that multiple neighbourhood characteristics were associated with alcohol use patterns in midlife, with associations more frequently observed in urban than in rural settings. Overall, heavy alcohol use was more prevalent in urban areas, while rural residents more often abstained from alcohol use. In urban areas, better transportation infrastructure, access to grocery stores, and a greater amount of rented housing were associated with heavy alcohol use, while higher greenness was associated with lower risk of heavy alcohol use. In contrast to urban areas, in rural areas a greater amount of rented housing was associated with a higher likelihood of abstinence. In both settings, a higher proportion of working-aged people and living in advantaged neighbourhoods were associated with a lower likelihood of abstinence. Most associations remained significant after adjusting for self-reported brisk and accelerometer-measured moderate-to-vigorous physical activity.

### Comparison with earlier studies

According to a previous systematic review, 18% of studies reported higher heavy or high-risk alcohol use in urban areas than in rural areas, as our study did, though most found the opposite [3]. Additionally, our findings regarding the link between improved transportation infrastructure (bus stops and pedestrian/cycle paths) and heavy alcohol use are partly in line with those of an earlier study [10]. However, in the study by Leung et al. [10], transportation infrastructure was assessed by the presence of subway lines and alcohol use as alcohol expenditure. Because Finland is a sparsely populated country, most towns outside the capital city area of Helsinki are too small [40, 41] to have subway lines; therefore, comparison between studies is difficult.

Our findings in urban areas regarding grocery stores, where alcoholic beverages can be purchased, are consistent with those of earlier studies; higher outlet density increased alcohol use, while greater distance reduced it [7–10]. Additionally, an earlier Finnish study found that a change in distance from home to the nearest off-premises alcohol outlet affected the risk of heavy alcohol use in women [42]. Another study showed that each extra off-premises alcohol outlet was associated with 3% increased risk of alcohol-related emergency department visits [43]. Dimova et al. [44] suggested that the physical availability of alcohol, proximity to local amenities, and late-night opening hours may be linked to the normalisation of alcohol use and permissive drinking environments. However, our study did not have information on the locations of alcohol-only retail stores. In Finland in 2014, all alcoholic beverages above 4.7% as well as distilled beverages over 2.8% were sold solely in monopoly stores [45]. The only exceptions were that Finnish farm wineries and sahti (a traditional Finnish ale) producers could sell their products with up to 13% alcohol content. Fermented beverages up to 4.7% were also sold in kiosks and petrol stations [45]. In addition, our results indicate that the number of restaurants was not related to alcohol use patterns in either urban or rural areas. This may be because the number of restaurants in our study also included facilities without alcohol licenses, such as canteens.

In this study, a higher proportion of rented housing was associated with heavy alcohol use in urban areas, but with a higher likelihood of abstinence in rural areas. This could be because in rural areas, renting may represent a different type of housing pattern; for instance, rental arrangements may be more short-term, and housing may be cheaper compared to urban areas. Another possibility is that among those living in rental housing in rural areas, there may be individuals whose lifestyle orientations or life philosophy do not include the use of alcohol. Building on this, other aspects of the physical environment also seem to influence drinking behaviour. In urban areas, residential greenness was associated with a lower likelihood of heavy alcohol use while greater distance to the nearest forest was associated with a higher likelihood. This may also reflect the overall urban structure, as more densely built urban areas tend to be less green and located farther from forests but may also have more stores where alcoholic beverages can be purchased. These findings partly mirror those of an earlier study among adolescents and young adults, which found that residential greenness reduced the odds of frequent binge drinking [13]. A green environment may reduce alcohol use by providing stress-relieving and well-being-enhancing experiences and increasing opportunities for healthy leisure activities.

Our finding that a higher proportion of working-aged people is associated with lower likelihood of abstinence is in line with previous research indicating that working-aged individuals consume most of the alcohol in Finland. Additionally, middle-aged people are most at risk of serious alcohol-related harm [4]. The finding that living in a more advantaged neighbourhood is associated with lower likelihood of abstinence is also in line with an earlier systematic review which revealed that 13.5% of studies found higher alcohol use is more common in affluent neighbourhoods [14]. The lower rates of abstinence in areas with more working-aged people and in more affluent residential areas may be attributed to the higher incomes. It has previously been shown that living in a lower-income household for a long time is related to a higher likelihood of abstinence and heavy alcohol use compared to light/moderate use [46]. Additionally, some people who are abstinent or light users may have multiple chronic conditions [47].

Our findings on physical activity suggest that accelerometer measures of moderate-to-vigorous physical activity may act to some extent as a confounding factor in the associations between neighbourhood characteristics and abstinence. When self-reported amount of brisk physical activity was included in the models, most associations remained robust, indicating that amount of self-rated brisk physical activity did not substantially affect the results. The observed discrepancies between self-reported and accelerometer-measured physical activity likely reflect differences in the coverage of these measurement approaches. Self-reported measures capture only leisure-time brisk physical activity, whereas accelerometer-based assessments capture physical activity throughout the day, including occupational and other non-leisure domains. Consequently, these methodological differences may contribute to variations in estimated activity levels and their associations with different outcomes.

Our results show that neighbourhood characteristics are more often related to alcohol use patterns in urban than in rural areas. This disparity may be cautiously attributed to the higher environmental exposure to alcohol, greater availability, easier access, and greater psychosocial stress in urban settings [48]. Our findings also emphasize the protective role of natural environments in urban areas, which is important when formulating public health strategies and building and shaping neighbourhoods.

Strengths and limitations of the study.

Our study has several strengths. As far as we know, this was the first population-based study to evaluate the relationships between objectively measured residential neighbourhood characteristics in both urban and rural areas including both self-reported and device-based measures of physical activity as potential confounding factors in alcohol use patterns. We were able to use large, representative population-based data and a comprehensive set of neighbourhood characteristics created with a Geographic Information System.

The limitations of this study include its cross-sectional design; we could not establish causal relationships between neighbourhood characteristics and alcohol use patterns in urban and rural areas. Because our alcohol use variable was skewed, we could not treat it as a continuous measure. Therefore, we classified participants as abstainers, moderate users, or heavy users [22], categories also used previously in the NFBC1966 study [21]. Another limitation is that some participants may have lived in their neighbourhood for longer than others. Furthermore, although we adjusted for important factors, other unmeasured factors may still have exerted confounding effects. The possibility of chance findings must also be considered. In addition, our study focused on middle-aged individuals, so the results may not be generalizable to other age groups. When comparing our results to other studies, it should be noted that definitions of urban and rural areas vary across countries, and other neighbourhood characteristics are also measured differently. In Finland, for example, towns are quite small, except for a few large cities which have hundreds of thousands of inhabitants [40]. Additionally, confidence intervals were wide concerning the association between the proportion of working-aged inhabitants and heavy use after adjustments in urban areas. Participation in the 46-year follow-up study was more common among women than among men. The participants were more likely to be employed, married, and to have children. Those with higher socioeconomic status and education levels were more likely to participate than their peers [20]. This may have affected our results.

## Conclusions

In conclusion, this population-based birth cohort study provides a comprehensive view of the associations between neighbourhood characteristics and alcohol use patterns in both urban and rural areas. Neighbourhood characteristics appear to be associated with alcohol use patterns largely independent of physical activity. These findings highlight the need to consider neighbourhood-level determinants in public health strategies aimed at reducing harmful alcohol consumption in middle-aged adults.

## Data Availability

NFBC data are available from the University of Oulu, Infrastructure for Population Studies. Permission to use the data can be applied for research purposes via an electronic material request portal. In the use of data, we follow the EU general data protection regulation (679/2016) and the Finnish Data Protection Act.The use of personal data is based on a cohort participant’s written informed consent in their latest follow-up study, which may cause limitations to its use. Please, contact the NFBC project center (NFBCprojectcenter(at)oulu.fi) and visit the cohort website (www.oulu.fi/nfbc) for more information.
